# Bipartite Structures in Social Networks: Traditional versus Entropy-Driven Analyses

**DOI:** 10.3390/e21030277

**Published:** 2019-03-13

**Authors:** Wilhelm Rödder, Andreas Dellnitz, Friedhelm Kulmann, Sebastian Litzinger, Elmar Reucher

**Affiliations:** 1 Department of Operations Research, FernUniversität in Hagen, 58097 Hagen, Germany; 2 Department of Quantitative Methods, FernUniversität in Hagen, 58097 Hagen, Germany; 3 Department of Business Administration, Private Hochschule für Wirtschaft und Technik, 49377 Vechta, Germany

**Keywords:** social network analysis, bipartite structures, directed graphs, attenuated links, entropy

## Abstract

A special type of social networks is the so-called affiliation network, consisting of two modes of vertices: actors and events. Up to now, in the undirected case, the closeness of actors in such networks has been measured by their jointly-attended events. Indirect contacts and attenuated and directed links are of minor interest in affiliation networks. These flaws make a veritable estimation of, e.g., possible message transfers amongst actors questionable. In this contribution, first, we discuss these matters from a graph-theoretical point of view. Second, so as to avoid the identified weaknesses, we propose an up-and-coming entropy-based approach for modeling such networks in their generic structure, replacing directed (attenuated) links by conditionals: if-then. In this framework, the contribution of actors and events to a reliable message transfer from one actor to another—even via intermediaries—is then calculated applying the principle of maximum entropy. The usefulness of this new approach is demonstrated by the analysis of an affiliation network called “corporate directors”.

## 1. Introduction

Social Networks (SN) are actors and their mutual relationships. The aim of Social Network Analysis (SNA) is to record and interpret structures within the social fabric. How do different actors or groups of actors act within networks? What benefits do they gain from this, and what disadvantages do they suffer in interacting with other actors? Ever since Moreno [1] published his ground-breaking work, scientists have been able to support their analyses with graphic representations of these networks. Actors are displayed as nodes and relationships as either undirected or directed edges (arrows). They are a visual representation of symmetrical or asymmetrical relationships. Such simple social structures are generalized in multigraphs or hypergraphs. Whereas multigraphs show several relationships, hypergraphs focus on so-called hyperconnections; meaning connections that link up more than two actors.

Social affiliation networks are a specific type of social network. The term *affiliation* here describes a known membership or affinity. In other words, social affiliation networks are used to describe which actors belong to which clubs, associations, or interest groups or whether they meet at specific events. Probably the best-known, frequently-cited example of this is that of the “Southern Women” [2]. The authors describe which society ladies in a small town in the Southern States of the U.S. attend what events, and these observations are subject to a profound sociological investigation of the town’s society. Such, among others, affiliation networks typically have two modes, making them bipartite. The actors constitute one type of network node, and the events the other type. Only elements with different node types can form relationships. Once such a network has been selected, the actors can be investigated in relation to the type and number of their contacts, and the clubs can be examined in relation to their attractiveness or suitability for exchanging messages, knowledge, etc. Deviating from classical notation, from now on, we use the expression *platform* instead of clubs, associations, or events. Platforms are the loci of message transfer, be it notice-boards, newsgroups, or social media. Mind the fact that in reality, message transfers via platforms do not necessarily involve personal contacts. Very frequently, personal contacts are replaced by media channels, as mentioned above.

Interestingly enough, there are several applications of bipartite graphical structures in other fields of research, as well, such as collaboration networks joining, e.g., projects and project partners [3,4] or bibliographic networks joining, e.g., authors and papers or conferences, respectively [5,6].

Borgatti and Everett [7], Borgatti [8] described bipartite structures and their special features: the affiliation matrix, the sociomatrix that can be deduced from this, and different ways to determine the “proximity” of actors as an indicator of message transfer between them. They elaborated on how traditional indexes, such as network centralization or the centralities of actors and platforms, may only ever be applied after undergoing significant changes and outlined the level of modification required. Clearly, the centralization of a bipartite network cannot be the same as that of a traditional network, since connections cannot exist between nodes of the same type. The authors further investigated how the key feature of a two-mode system impacts the cohesive groups within networks, on structural and regular equivalence, and on many others.

One aspect that all traditional analyses of affiliation networks have in common is that links are always viewed as undirected. Their semantics consists of the non-directional connection between actors and platforms.

Therefore, such structures are only ever evaluated and analyzed in relation to the number of such links and the parameters resulting from this. “How often does actor *i* meet actor *j* at different clubs or associations?” “How many of the members of club *k* are also members of club *l*?” This is then used to determine the likelihood that, e.g., a message will be forwarded from *i* to *j* or to deduce the social proximity between *k* and *l*. Instead of focusing merely on certain transfers of messages or news, we also accept an attenuated version. The idea of an *attenuated* transfer was first mentioned by Katz [9] and referenced and developed further by Bonacich [10] and then by Bonacich and Lloyd [11]. Everett took up this idea and applied it to undirected affiliation networks [12]. The present paper makes several generalizations:

- It focuses not on undirected, but instead on directed bipartite graphs. This takes into account the simple fact that, e.g., a message transfer from an actor to a platform does not necessarily occur with the same level of likelihood as vice versa. None of the quoted authors considered attenuated *and* directed affiliation networks.
- Rödder et al. [13,14] studied an initial example of modeling general social networks using the principle of entropy. We will apply such probabilistic modeling in order to analyze attenuated directed social affiliation networks effectively. Such analysis then permits rankings of actors and platforms with respect to their influential power.

The Introduction is followed by Section 2, which outlines traditional affiliation network analysis, to provide a basis for presenting and defining the new method in subsequent sections. Section 2.1 formally presents affiliation networks, whereas Section 2.2 sets out direct and indirect contact frequencies between actors. Section 2.3 then outlines the transfer probabilities for messages that can be deduced based on the frequency of contact according to selected normalizations. A short example is then presented, and relating questions are addressed. Section 2.4 continues by generalizing to directed graphs. Section 3 is dedicated to the novel modeling method: Section 3.1 focuses on syntax and semantics and Section 3.2 on how to set up a probabilistic model for a bipartite network. Section 4 shows the potential of an entropy-based model by applying it to a network of 20 actors and 24 platforms. Finally, Section 5 offers a summary and attempts to present the prospects for further research.

## 2. Affiliation Networks and Traditional Analyses

### 2.1. Basic Concepts and Their Sociological Meanings

As outlined in the Introduction, affiliation networks have two types of entities: actors and platforms. Some actors share specific platforms: they might, for instance, go to the same clubs, use the same social media channels, etc., while others might not. The sociological literature consistently assumes that the frequency of joint platforms amongst actors supports their willingness to exchange knowledge or messages.

A (general) graph consists of a set of nodes V and a set of edges E: G=(V,E). If two nodes v,w∈V are linked by an edge e∈E, they are called adjacent. If e is undirected, we write e=(v,w). If it is directed, then e=〈v,w〉; the direction in the latter case is from v to w. The sociological context of such graphs is well known; see Scott [15].

**Figure 1:**
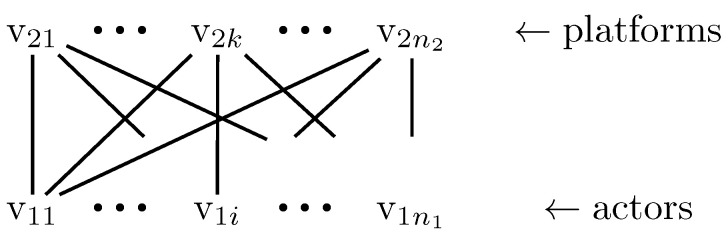
Generic undirected affiliation network.

A graph G=(V,E) is bipartite if $V=V_{1}\dot{\cup }V_{2},V_{1}\neq \emptyset ,V_{2}\neq \emptyset$, and the following applies for each connection (v,w), or 〈v,w〉: if $v\in V_{1}$, then $w\in V_{2}$ and vice versa. Nodes from $V_{1}$ are never mutually linked, and neither are nodes from $V_{2}$. The cardinalities of these node sets are $n_{1}=\left|V_{1}\right|$ and $n_{2}=\left|V_{2}\right|$. We will start by focusing on undirected bipartite graphs, typically used to describe affiliation networks. We will use $V_{1}$ to describe the actors and $V_{2}$ to describe the platforms. Figure 1 shows a generic affiliation network.

The first index of the nodes shows the entity type, and the second numbers them sequentially.

### 2.2. Contact Frequencies between Actors in Undirected Graphs

In an affiliation matrix for a network described in Section 2.1, rows will denote actors and columns will denote platforms; it has entry one in row $v_{1i}$ and column $v_{2k}$ if $(v_{1i},v_{2k})\in E$ and zero, otherwise. This affiliation matrix $A=A_{n_{1}\times n_{2}}$ hence is a rectangular matrix with $n_{1}$ rows and $n_{2}$ columns. Therefore, ${\mathrm{AA}}^{T}$ is an $n_{1}\times n_{1}$ matrix, whose entries are the actors’ mutual contact frequencies. In the same way, $A^{T}A$ is an $n_{2}\times n_{2}$ matrix, whose entries constitute the number of joint memberships in platforms.

Borgatti [8], Everett [12] showed a block matrix that combines both views, calling this a “bipartite adjacency matrix B”.

(1) $$\begin{array}{c} B=\left(\begin{array}{cc} 0_{n_{1}\times n_{1}} & A_{n_{1}\times n_{2}}\\ A_{n_{2}\times n_{1}}^{T} & 0_{n_{2}\times n_{2}} \end{array}\right)\\ \mathrm{Key}:\;\mathrm{Zero}\;\mathrm{refers}\;\mathrm{to}\;\mathrm{matrices}\;\mathrm{filled}\;\mathrm{with}\;\mathrm{zero}\;\mathrm{entries}. \end{array}$$

B is square $\left(n_{1}+n_{2}\right)\times \left(n_{1}+n_{2}\right)$. B·B then gives:

(2) $$\begin{array}{c} B^{2}=\left(\begin{array}{cc} AA^{T} & 0\\ 0 & A^{T}A \end{array}\right) \end{array}$$

and consequently, both the actors’ contact frequencies and joint memberships in platforms. $B^{3}$, $B^{4}$, as well as greater powers can be formed directly, and this, e.g., gives:

(3) $$\begin{array}{cc} B^{3} & =\left(\begin{array}{cc} 0 & AA^{T}A\\ A^{T}AA^{T} & 0 \end{array}\right) \end{array}$$

(4) $$\begin{array}{cc} B^{4} & =\left(\begin{array}{cc} AA^{T}AA^{T} & 0\\ 0 & A^{T}AA^{T}A \end{array}\right). \end{array}$$

While the entries in $B^{2}$ show the actors’ contact frequencies and joint memberships in platforms (see above), $B^{3}$ demonstrates the impossibility of linking actors with actors or platforms with platforms in three steps. Finally, $B^{4}$ shows the contact frequencies between actors also using an intermediary and indirect joint memberships. In other words, actor *i* might not be a member of the same platform as actor *j*, but both have the same membership as a third party. This can now be applied to platforms and extrapolated to matrices for greater powers.

### 2.3. From Contact Frequencies to Transfer Probabilities

If we assume that the contact frequencies between actors indicate their disposition towards passing on messages or news, this would be in line with the basic idea put forward by the respective literature [7,8,16]. We will now present a short example to make this idea more transparent.

**Figure 2:**
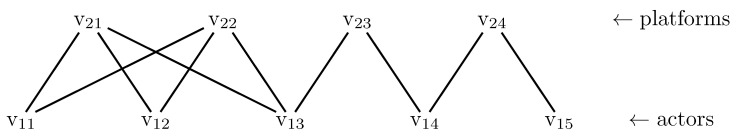
A concrete affiliation network.

Let us take a look at the affiliation network in Figure 2.

We chose this example because:

(i) it includes actors not present in all platforms
(ii) some pairs of actors share several platforms
(iii) some pairs of actors can only contact each other via an intermediary
(iv) some pairs of actors can only contact each other via two intermediaries.

These verbal characterizations can now be visualized in matrices, with explanations provided.

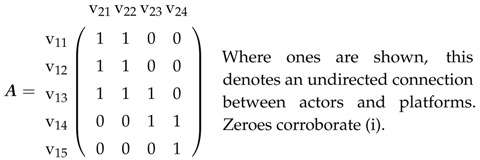
(5)

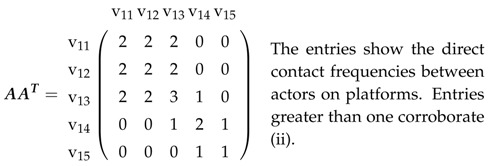
(6)

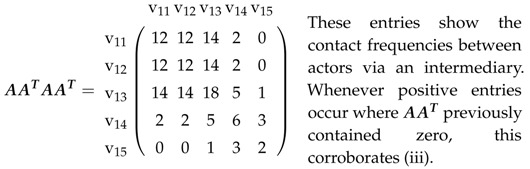
(7)

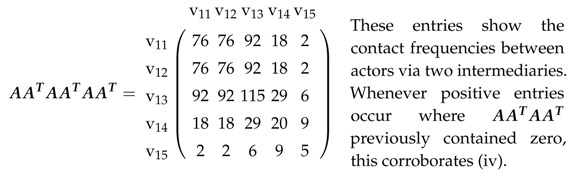
(8)

The traditional literature only uses direct contacts such as those contained in the matrix ${\mathrm{AA}}^{T}$ to calculate transfer probabilities. Such probabilities are estimated based on relative frequencies applying various scaling rules. If ${\left(u_{ij}\right)}_{n_{1}\times n_{1}}$ are the elements of the matrix ${\mathrm{AA}}^{T}$, then we can scale as follows according to Borgatti [8]:

(9) $$\begin{array}{c} \frac{u_{ij}}{n_{2}} \end{array}$$

(10) $$\begin{array}{c} \frac{u_{ij}}{\min(u_{ii},u_{jj})} \end{array}$$

(11) $$\begin{array}{c} \frac{u_{ij}}{u_{ii}+u_{jj}-u_{ij}} \end{array}$$

With reference to Formulas (9)–(11) and ${\mathrm{AA}}^{T}$ as per (6), we obtain the following transfer probabilities:

(9′) $$\left(\begin{array}{ccccc} - & 0.5 & 0.5 & 0 & 0\\ 0.5 & - & 0.5 & 0 & 0\\ 0.5 & 0.5 & - & 0.25 & 0\\ 0 & 0 & 0.25 & - & 0.25\\ 0 & 0 & 0 & 0.25 & - \end{array}\right)$$

(10′) $$\left(\begin{array}{ccccc} - & 1 & 1 & 0 & 0\\ 1 & - & 1 & 0 & 0\\ 1 & 1 & - & 0.5 & 0\\ 0 & 0 & 0.5 & - & 1\\ 0 & 0 & 0 & 1 & - \end{array}\right)$$

(11′) $$\left(\begin{array}{ccccc} - & 1 & 0.67 & 0 & 0\\ 1 & - & 0.67 & 0 & 0\\ 0.67 & 0.67 & - & 0.25 & 0\\ 0 & 0 & 0.25 & - & 0.5\\ 0 & 0 & 0 & 0.5 & - \end{array}\right)$$

The missing entries along the diagonal are self-explanatory.

All scalings can be applied easily, but nevertheless, the question arises which of these transfer probabilities should be used in further analyses.

Using the examples of (7) and (8), this section demonstrates that indirect contacts can exist via one, two, or several intermediaries. Similar transfer probability patterns could also easily be estimated from ${\mathrm{AA}}^{T}{\mathrm{AA}}^{T}$ or ${\mathrm{AA}}^{T}{\mathrm{AA}}^{T}{\mathrm{AA}}^{T}$, but the problem of how to choose the most suitable scaling would remain.

Keep in mind that the entries in the matrices (6)–(8) describe walks of lengths of 2, 4, or 6 and that this might involve multiple contacts between nodes or repeated runs along the same edge. By way of example, from the 12 walks from $v_{11}$ to $v_{12}$ in (7), four come back to $v_{11}$ before reaching $v_{12}$; and 10 are not even trails, as they run twice along the same edge. We verify that there are only two paths from $v_{11}$ to $v_{12}$ with one intermediary, in this case, $v_{13}$. What finally remains as a suitable set of direct or indirect contacts is an open question and is highly context-dependent.

If we accept that the messages can also be transferred indirectly via intermediaries, we must still determine how direct and indirect contacts or contact frequencies should be weighted in relation to each other. What is the significance of a contact via one or several intermediaries compared to a direct contact?

### 2.4. Contact Frequencies and Transfer Probabilities in Directed Graphs

The bipartite adjacency matrix—if modified slightly—is also ideal for representing directed graphs. Why directed graphs? As already mentioned in the Introduction, a message transfer from an actor to a platform does not necessarily occur with the same level of likelihood as vice versa. Imagine a club has set up a newsletter to inform its members about upcoming fundraising campaigns or future meetings of members, etc. Undoubtedly, the frequency of reaching a member when sending a message is different from the likelihood of an actor to reveal an item of information to the club. Hence, directed graphs are a must in affiliation network analysis. The upper part of (12) shows the adjacencies of actors in relation to platforms, while the lower part describes that of platforms to actors. In general, such matrices are not transposed with each other, of course. We can write the general version as:

(12) $$\begin{array}{c} \bar{B}=\left(\begin{array}{cc} 0 & A\\ \bar{A} & 0 \end{array}\right). \end{array}$$

$\bar{B}$ again is square $\left(n_{1}+n_{2}\right)\times \left(n_{1}+n_{2}\right)$. $\bar{B}\cdot \bar{B}$ now results in:

(13) $$\begin{array}{c} {\bar{B}}^{2}=\left(\begin{array}{cc} A\bar{A} & 0\\ 0 & \bar{A}A \end{array}\right). \end{array}$$

In addition, ${\bar{B}}^{3}$, ${\bar{B}}^{4}$, and greater powers can also be formed, which, e.g., creates:

(14) $$\begin{array}{cc} {\bar{B}}^{3} & =\left(\begin{array}{cc} 0 & A\bar{A}A\\ \bar{A}A\bar{A} & 0 \end{array}\right) \end{array}$$

(15) $$\begin{array}{cc} {\bar{B}}^{4} & =\left(\begin{array}{cc} A\bar{A}A\bar{A} & 0\\ 0 & \bar{A}A\bar{A}A \end{array}\right). \end{array}$$

The entries in ${\bar{B}}^{2}$ again show the contact frequencies between actors (upper part) and joint memberships (lower part). ${\bar{B}}^{3}$ and ${\bar{B}}^{4}$ can be interpreted accordingly; for details, see Section 2.2. These frequencies are of course significantly limited by the direction of arrows.

**Figure 3:**
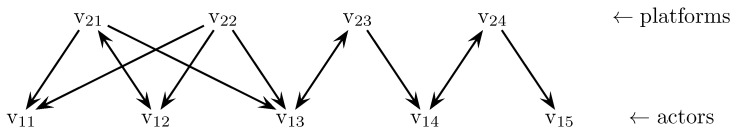
A concrete directional affiliation network.

Figure 3 contains all connections from platforms to actors as in Figure 2, now directed, but it only has three connections from actors to platforms. We can now specify A, $\bar{A}$, $A\bar{A}$, $\bar{A}A$, $A\bar{A}A\bar{A}$, $\bar{A}A\bar{A}A$.

(16) $$\begin{array}{cccc} A & =\left(\begin{array}{cccc} 0 & 0 & 0 & 0\\ 1 & 0 & 0 & 0\\ 0 & 0 & 1 & 0\\ 0 & 0 & 0 & 1\\ 0 & 0 & 0 & 0 \end{array}\right) & \bar{A} & =\left(\begin{array}{ccccc} 1 & 1 & 1 & 0 & 0\\ 1 & 1 & 1 & 0 & 0\\ 0 & 0 & 1 & 1 & 0\\ 0 & 0 & 0 & 1 & 1 \end{array}\right) \end{array}$$

(17) $$\begin{array}{cccc} A\bar{A} & =\left(\begin{array}{ccccc} 0 & 0 & 0 & 0 & 0\\ 1 & 1 & 1 & 0 & 0\\ 0 & 0 & 1 & 1 & 0\\ 0 & 0 & 0 & 1 & 1\\ 0 & 0 & 0 & 0 & 0 \end{array}\right) & \bar{A}A & =\left(\begin{array}{cccc} 1 & 0 & 1 & 0\\ 1 & 0 & 1 & 0\\ 0 & 0 & 1 & 1\\ 0 & 0 & 0 & 1 \end{array}\right) \end{array}$$

(18) $$\begin{array}{cccc} A\bar{A}A\bar{A} & =\left(\begin{array}{ccccc} 0 & 0 & 0 & 0 & 0\\ 1 & 1 & 2 & 1 & 0\\ 0 & 0 & 1 & 2 & 1\\ 0 & 0 & 0 & 1 & 1\\ 0 & 0 & 0 & 0 & 0 \end{array}\right) & \bar{A}A\bar{A}A & =\left(\begin{array}{cccc} 1 & 0 & 2 & 1\\ 1 & 0 & 2 & 1\\ 0 & 0 & 1 & 2\\ 0 & 0 & 0 & 1 \end{array}\right) \end{array}$$

The estimation of transfer probabilities from contact frequencies follows the same logic as in Section 2.3 and results in similar difficulties as for non-directional bipartite structures. Therefore, this is omitted here.

To sum up, this section shows a severe problem in determining transfer probabilities by means of direct or indirect contact frequencies, be the respective graphs undirected or directed. Due to the great variety of calculating such probabilities, the analyst might choose the wrong aggregation method and hence obtain a biased result. To the best of our knowledge, the only probabilistic method that leads to an unbiased representation of contact frequencies uses the principle of maximum entropy. All available data concerning direct and indirect contact frequencies are “married” in a probabilistic conditional-logical framework; for an axiomatic justification, cf. Kern-Isberner [17].

## 3. Entropy-Driven Bipartite Network Analysis

### 3.1. Syntax and Network Load

The details in this section are based on the representation of knowledge processing in social networks in Rödder et al. [13], Brenner et al. [18]. They are repeated here and applied to affiliation networks in the subsequent Section 3.2.

Let us take a set of *n* nodes $\left\{v_{1},\ldots ,v_{n}\right\}$. Each node $v_{i}$ is represented by a binary variable $V_{i}$ with the values $V_{i}=v_{i}$ and $v_{i}=1/0$. Therefore, $v=\{v_{1},\ldots ,v_{n}\}$ are the respective configurations. For pairs of nodes, $V_{j}=1|V_{i}=1$ are conditionals; ∣ is the conditional operator. For a detailed discussion on conditionals, see, e.g., Calabrese [19] or also Rödder et al. [13].

The semantics of these symbols is as follows: $V_{i}=1/0$ is the proposition; node $v_{i}$ either knows the message (1) or not (0). The conditionals describe potential transfer: if $v_{i}$ has the message, then it probably has $v_{j}$. Therefore, conditionals replace weighted arrows in graphs.

Let us assume that sociological inquiries have only provided transfer probabilities $p_{ij}$ for several pairs of nodes $\langle v_{i},v_{j}\rangle$, and not for others. As such, this network consists of a set N⊆{1,…,n}×{1,…,n}, and the related conditionals and probabilities:

(19) $$V_{j}=1|V_{i}=1\;\mathrm{with}\;p_{ij}\;\mathrm{for}\;\left(i,j\right)\in N.$$

Now, we look for a probability distribution Q on {v}, which takes account of the transfer probabilities:

(20) $$Q\left(V_{j}=1|V_{i}=1\right)=p_{ij}\;\mathrm{for}\;\left(i,j\right)\in N.$$

Such a distribution is called a *network load*.

If $p_{ij}$ are entered consistently, then (21) yields a particular distribution on a network.

(21) $$\begin{array}{c} Q^{*}=\arg\min R\left(Q,P^{0}\right)=\sum_{v}Q\left(v\right)\log_{2}\frac{Q(v)}{P^{0}\left(v\right)}\\ \mathrm{subject}\;\mathrm{to}\;Q\left(V_{j}=1|V_{i}=1\right)=p_{ij},\;\left(i,j\right)\in N. \end{array}$$

Equation (21) respects all $p_{ij}$ and creates the distribution $Q^{*}$ of Minimal Relative Entropy (MinREnt) or Kullback–Leibler divergence *R* from the uniform distribution $P^{0}$ on {v}. As is well known, the minimization in (21) is equivalent to the maximization of the entropy $H=-\sum_{v}Q\left(v\right)\log_{2}Q\left(v\right)$. Therefore, we call $Q^{*}$ a Maximum Entropy (MaxEnt) load on the net. $Q^{*}$ is a distribution used in artificial intelligence as a knowledge base for the entire network structure [14]. Keep in mind that (21) has a strict axiomatic justification; see again Kern-Isberner [17].

For solving optimization Problem (21), an algorithmic framework is needed. Two frameworks are, e.g., LEXMED [20] and SPIRIT [21]. In the remainder of this paper, we focus on the latter, which was developed at the FernUniversität in Hagen, Germany. For its functionalities, see [21].

Once a distribution is calculated, what is the impact of a specific message sent by node $v_{i}$: $V_{i}:=1$? If node $v_{i}$ sends the message, it will penetrate the network according to the probabilistic conditional structure. This process can be performed in SPIRIT [22] by solving the equation:

(22) $$\begin{array}{c} Q^{**}=\arg\min R\left(Q,Q^{*}\right)=\sum_{v}Q\left(v\right)\log_{2}\frac{Q(v)}{Q^{*}\left(v\right)}\\ \mathrm{subject}\;\mathrm{to}\;Q(V_{i}=1)=1. \end{array}$$

$Q^{**}$ is the distribution on the network of minimum divergence from $Q^{*}$ subject to the condition that $V_{i}=1$. Therefore, this means conditioning a distribution as a whole. If we have $Q^{**}$, then $Q^{**}\left(V_{j}=1\right)$ can be calculated for other j≠i. $Q^{**}\left(V_{j}=1\right)$ is the probability that $v_{j}$ receives the message if $v_{i}$ sends it, i.e., actor *j*’s reception probability. According to the considerations in this section, $v_{j}$ can be a neighbor of $v_{i}$, or not. $Q^{**}\left(V_{j}=1\right)$ even applies to $v_{j}$, which can only be reached in the network via one or several intermediaries. Rödder et al. [23] also dealt with the extent to which such reception probabilities are only vague conjectures or resilient estimates.

The probabilistic conditional-logical framework developed so far will be applied to two-mode networks in the next section.

### 3.2. MaxEnt Distributions in Two-Mode Networks

Now, we consider the sets $V_{1}=\left\{v_{11},\ldots ,v_{1i},\ldots ,v_{1n_{1}}\right\},\;V_{2}=\left\{v_{21},\ldots ,v_{2k},\ldots ,v_{2n_{2}}\right\}$ and the related variables $\left\{V_{11},\ldots ,V_{1i},\ldots ,V_{1n_{1}}\right\},\;\left\{V_{21},\ldots ,V_{2k},\ldots ,V_{2n_{2}}\right\}$.

Conditionals can then be displayed as follows:

- $V_{1i}=1|V_{2k}=1\;\left[p_{ki}\right]$ for transfers from platforms to actors and
- $V_{2k}=1|V_{1i}=1\;\left[p_{ik}\right]$ for transfers from actors to platforms,

with the probabilities $p_{ki}$ and $p_{ik}$.

**Figure 4:**
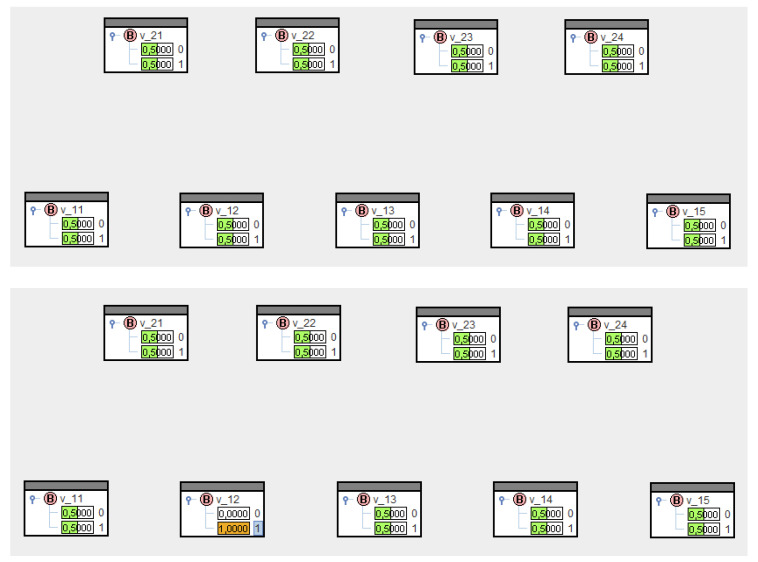
Marginal distributions for an empty set of conditionals before and after having evidentiated.

Let us refer to Figure 3 to illustrate the relationships, and assume, first, that no transfer probabilities are known. Therefore, the set of conditionals in (21) is empty, and according to Figure 4 (top), the set of all nodes yields the marginal distributions $P^{0}\left(V_{1i}=1\right)=P^{0}\left(V_{2k}=1\right)=0.5$. The conditioning process, i.e., solving Equation (22) for specific nodes, can be realized in SPIRIT through clicking, e.g., $V_{12}:=1$ results in Figure 4 (bottom). As expected, the marginal distributions for all nodes—except $V_{12}$—have not changed.

Exemplary probabilities are now assigned to the conditionals as shown in Table 1.

This can be depicted compactly as matrices of transfer probabilities:

(23) $$\begin{array}{c} P=\left(\begin{array}{cccc} 0 & 0 & 0 & 0\\ 0.8 & 0 & 0 & 0\\ 0 & 0 & 0.8 & 0\\ 0 & 0 & 0 & 0.8\\ 0 & 0 & 0 & 0 \end{array}\right)\;\bar{P}=\left(\begin{array}{ccccc} 1 & 1 & 1 & 0 & 0\\ 1 & 1 & 1 & 0 & 0\\ 0 & 0 & 1 & 1 & 0\\ 0 & 0 & 0 & 1 & 1 \end{array}\right) \end{array}$$

**Figure 5:**
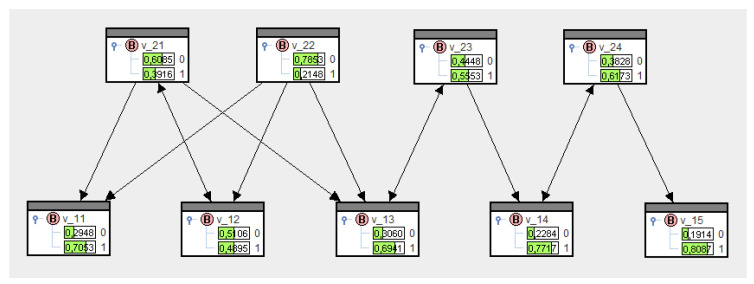
A bipartite network in SPIRIT.

These probabilities mean that the transfer from platforms to actors is certain (=1), whereas actors are less likely to communicate (=0.8). If we enter the conditionals and these transfer probabilities in SPIRIT, then upon solving Equation (21), we come up with $Q^{*}$, whose marginal probabilities are shown in the variables in Figure 5.

Now, in contrast to Figure 4 (top), the marginal distributions have changed. They enable us to make a priori estimates of message transfers in the network, merely based on the structure. We notice lower probabilities for V=1 for nodes more likely to be senders and higher probabilities for V=1 for nodes more likely to be receivers. A first attempt to make these observations more transparent are the following information-theoretical considerations.

For V:=1 as in (22), $R(Q^{**},Q^{*})$ measures the change of the conditional structure from $Q^{*}$ to $Q^{**}$, cf. Brenner et al. [18]. Rödder et al. [13] called this number the diffusion potential of a node, and in Theorem 2 on page 7975, it is shown that:

(24) $$\begin{array}{c} R\left(Q^{**},Q^{*}\right)=-\log_{2}Q^{*}\left(V=1\right). \end{array}$$

The entire conditional structure change in the network is already anticipated in (24).

Therefore, $-\log_{2}\left(Q^{*}\left(V_{1i}=1\right)\right)$ is the measure of diffusion in the network for each actor *i*. The deeper his/her message penetrates the network, the higher his/her diffusion.

$-\log_{2}\left(Q^{*}\left(V_{2k}=1\right)\right)$ is also the measure of diffusion for platform *k* in the network. The deeper a message known there penetrates the network—through direct or indirect contacts—the higher is its diffusion potential.

The greater $-\log_{2}Q^{*}\left(V=1\right)$, the smaller is $Q^{*}\left(V=1\right)$ for each node in the network; either for an actor or a platform. The MaxEnt distribution assigns low probabilities to related V=1 correctly, whose network penetration—direct or indirect contacts to other nodes—is high. In the same way, it assigns high probabilities to nodes with low network penetration.

**Figure 6:**
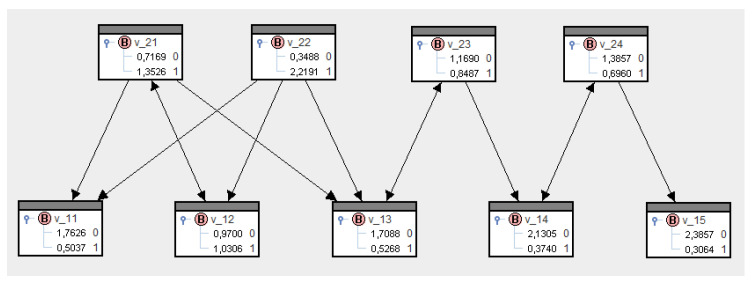
Information values for Figure 5.

SPIRIT allows marginal probabilities to be switched to negative logarithms. Figure 6 shows these logarithms for Figure 5. As such, $V_{12}=1$ has the greatest diffusion potential of all actors, and $V_{22}=1$ has the greatest diffusion potential of all platforms; this is in line with the intuitive conditional structure of the network.

**Figure 7:**
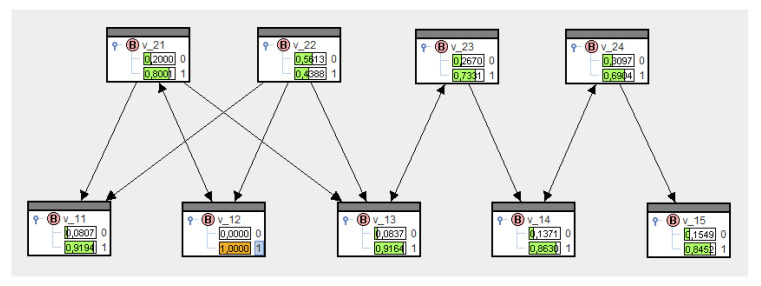
Conditional probabilities of reception under $V_{12}=1$.

**Figure 8:**
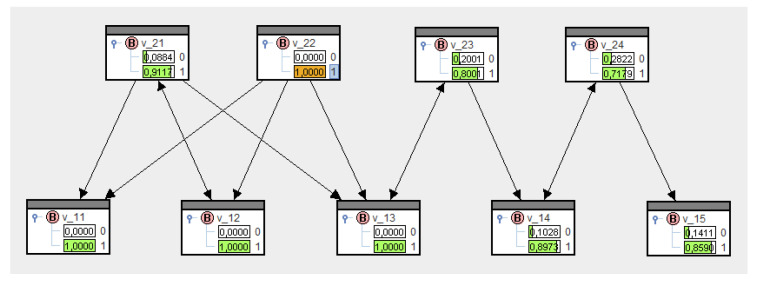
Conditional probabilities of reception under $V_{22}=1$.

Going back to the example in Table 1 and now clicking on the value 1 for the actor i=2 or the platform k=2, we get the probabilities of reception as shown in Figure 7 and Figure 8 for all actors and platforms. Obviously, they confirm the values of preset probabilities. They also demonstrate that, if actor i=2 sends the message, this also increases the probability of reception for actors with only indirect contacts. Therefore, the knowledge processing concept implemented in SPIRIT incorporates impacts on the probabilities of reception via one or several intermediaries.

The conditional probabilities of reception as presented in Figure 7 and Figure 8 are unbiased estimates of reception opportunities for all actors and platforms after the message has been sent. For instance, the probabilities for $V_{21}=1|V_{12}=1\;\left[0.8\right]$ in Figure 7 or for $V_{11}=1|V_{22}=1\;\left[1.0\right]$ in Figure 8 are preassigned, and other conditional probabilities of reception result from the MinREnt model, as in (22). Furthermore, we notice that the probability of reception for $V_{24}=1$ has only increased slightly compared to the a priori probability; see Figure 5 and Figure 7.

The next section examines how the concepts developed so far can be used to analyze a real medium-sized network.

## 4. Analysis of the Network “Corporate Directors”

**Figure A1:**
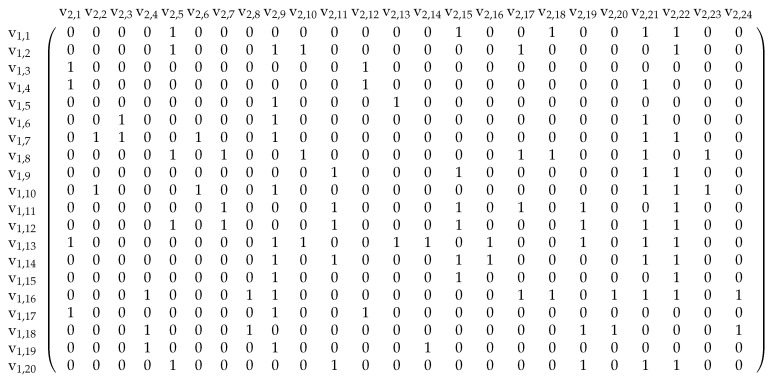
Affiliation matrix of the network “corporate directors”.

Barnes and Burkett [24] described an affiliation network called “corporate directors”. As the name suggests, this is a group of—in this case—20 directors and their memberships in 24 different institutions, such as clubs, management boards, supervisory boards, etc. The 99 affiliations are presented in the affiliation matrix in Figure A1 in Appendix A.

**Figure 9:**
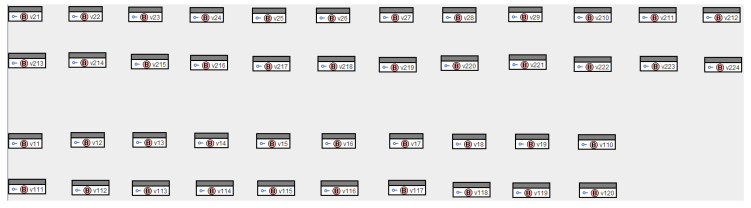
Bipartite structure of corporate directors in SPIRIT.

The preliminary column shows directors $\hat{=}$ actors and the preliminary row shows institutions $\hat{=}$ platforms. For reasons of consistency, we will call the directors $v_{1,1}$–$v_{1,20}$ and the institutions $v_{2,1}$–$v_{2,24}$. Figure 9 clearly shows the bipartite structure in SPIRIT.

The aim of this section is to demonstrate the usefulness of the entropy-driven analysis with respect to the transfer of messages and knowledge between actors and/or institutions. As in Section 3.2, we assume a directional bipartite structure with the following properties:

- Message transfer in the direction from institution to director is highly probable. Platforms are set up in order to make messages and news available to its members, where possible. As already stated in the Introduction, such message transfer might be realized via notice-boards, newsgroups, or social media. For our purposes, we choose the respective transfer probabilities to be a fictitious 0.9. Statistical analysis might help to verify such a 90% page view rate.
- The probabilities of message transfer from actors to institutions are even more difficult to survey due to the unknown willingness of persons to share information with others. We thwart this flaw using random numbers between 0.5 and one for the transfer probabilities. A first step to predicting the posting behavior of individuals the reader might find in Kim et al. [25].

**Figure 10:**
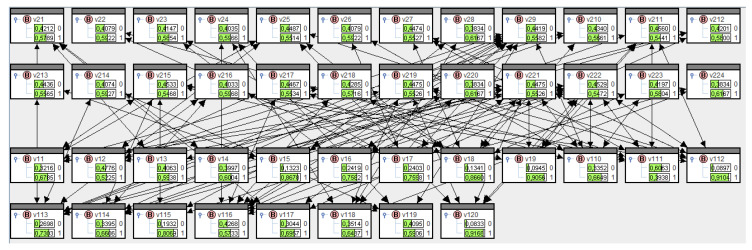
Marginal distribution across institutions and directors in SPIRIT.

**Figure 11:**
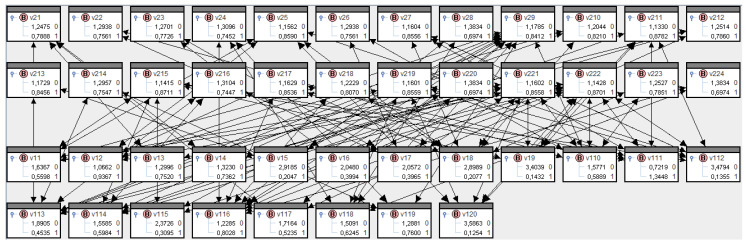
Information theory perspective in relation to institutions and directors in SPIRIT.

These transfer probabilities are entered into SPIRIT in a two-step process: first, for institutions to directors, then for directors to institutions. The conditionals for the former part can be deduced from adjacencies in Figure A1 in Appendix A plus 0.9 probability. The conditionals for the latter part are shown in Table A1 in Appendix A. Rödder et al. [21] set out in sufficient detail how this two-step learning process in SPIRIT can be implemented. The results are summarized in Figure 10, and the perspective according to information theory—diffusion—introduced in Section 3.2 and Equation (24) is shown in Figure 11.

As expected, diffusions in institutions tend to be greater than for directors, but some directors are also fairly communicative, e.g., directors $v_{1,11}$ and $v_{1,2}$.

The results in Figure 11 allow us to rank actors and institutions based on their diffusion. Here, high numbers mean much influence in the network, and low numbers mean less influence. Let us look at two actors and two institutions to further demonstrate the network analysis made possible using this model. Actor $v_{1,11}$ has the highest diffusion and actor $v_{1,20}$ the lowest. The lowest and highest diffusions for institutions are those for $v_{2,11}$ and, e.g., $v_{2,20}$; please refer back to Figure 11. If we now click on the respective values V=1 for these nodes, this means—as explained in detail above—that the message has been sent throughout the entire network.

Table 2 and Table 3 allow comparing the prior distribution to those after message posting.

His/her transfer probabilities make actor $v_{1,11}$ an opinion leader in the network. He/she reaches all other actors and raises their probability of reception significantly. This is entirely different for actor $v_{1,20}$. Now, the probabilities of reception are only minimally above a priori probabilities, which indicates only marginal influence in the network. The conditionals in Table A1 might help to clarify this issue.

As was already made apparent by the diffusions for institutions, we do not expect a strong heterogeneity with regard to message transfer. The examples of the two institutions $v_{2,11}$ and $v_{2,20}$ confirm this. Nevertheless, in most cases, we can see a clear increase in the probabilities of reception compared to those of the prior distribution in the net.

To summarize, the entropy-driven approach opens up new possibilities for analysis, which were not previously available in traditional graph-based methods.

## 5. Summary and Prospects

This paper considered social affiliation networks. The respective graphs have a bipartite structure; the node set is bi-modal: e.g., actors and clubs. First, we present the traditional approach towards analyzing such networks; this is based on the frequencies of mutual affiliations between actors and joint memberships in clubs. Indirect connections between actors—intermediaries—and indirect connections between clubs—an intermediary club might have members from either side—are formulated mathematically, and their potential for analysis is studied. Then, directed bipartite structures are depicted mathematically and differentiated from undirected structures.

A new type of probabilistic-conditional modeling is ideally suited to analyzing directional bipartite networks. Even *weighted* directional edges can be formulated as probabilistic conditionals. If an actor or a club is aware of a message or has specific knowledge, then he/she or it transfers it with preset probabilities. An entropy-driven information processing grounded in artificial intelligence supports such analyses. Even actors or clubs not in direct contact with the sender of such a message receive it via intermediaries. The software SPIRIT allows for calculation of respective transfer probabilities. The power of the new model is demonstrated analyzing a well-known example called “corporate directors”. The underlying network counts 20 directors and their memberships in 24 institutions. The new method permits a ranking of actors, as well as clubs with respect to their influential power. This kind of analysis is applicable to any bipartite network structure.

There are interesting prospects for further research on this issue:

- What are the consequences for the whole network if actors or groups of actors disappear (due to disease or career change)?
- Might indices like centrality and centralization suitably be defined in entropy-driven analyses of bipartite social networks?
- Can these analyses also apply to more complex structures like multigraphs or hypergraphs?

We hope that articles on these topics might stimulate our research. 

## Figures and Tables

**Table 1:** A specific affiliation network.

$\begin{array}{ccc} V_{11}=1|V_{21}=1\;\left[1.0\right] & V_{11}=1|V_{22}=1\;\left[1.0\right] & V_{12}=1|V_{21}=1\;\left[1.0\right]\\ V_{12}=1|V_{22}=1\;\left[1.0\right] & V_{13}=1|V_{21}=1\;\left[1.0\right] & V_{13}=1|V_{22}=1\;\left[1.0\right]\\ V_{13}=1|V_{23}=1\;\left[1.0\right] & V_{14}=1|V_{23}=1\;\left[1.0\right] & V_{14}=1|V_{24}=1\;\left[1.0\right]\\ V_{15}=1|V_{24}=1\;\left[1.0\right] & V_{21}=1|V_{12}=1\;\left[0.8\right] & V_{23}=1|V_{13}=1\;\left[0.8\right]\\ V_{24}=1|V_{14}=1\;\left[0.8\right] \end{array}$

**Table 2:** A priori probabilities and probabilities of message reception for actors with evidence $v_{1,11}$ and $v_{1,20}$.

$\begin{array}{cccccccccccc} & v_{1,1} & v_{1,2} & v_{1,3} & v_{1,4} & v_{1,5} & v_{1,6} & v_{1,7} & v_{1,8} & v_{1,9} & v_{1,10} & \ldots \\ A\;\mathrm{priori} & 0.68 & 0.52 & 0.59 & 0.60 & 0.87 & 0.76 & 0.76 & 0.87 & 0.91 & 0.66 & \ldots \\ \mathrm{Ev}.v_{1,11} & 0.98 & 0.93 & 0.82 & 0.89 & 0.90 & 0.95 & 0.97 & 0.98 & 0.90 & 0.94 & \ldots \\ \mathrm{Ev}.v_{1,20} & 0.69 & 0.53 & 0.60 & 0.61 & 0.87 & 0.76 & 0.76 & 0.87 & 0.91 & 0.67 & \ldots \\ & v_{1,11} & v_{1,12} & v_{1,13} & v_{1,14} & v_{1,15} & v_{1,16} & v_{1,17} & v_{1,18} & v_{1,19} & v_{1,20}\\ A\;\mathrm{priori} & 0.39 & 0.91 & 0.73 & 0.66 & 0.81 & 0.57 & 0.70 & 0.65 & 0.59 & 0.92\\ \mathrm{Ev}.v_{1,11} & - & 0.97 & 0.97 & 0.99 & 0.98 & 0.95 & 0.94 & 0.96 & 0.89 & 0.94\\ \mathrm{Ev}.v_{1,20} & 0.40 & 0.91 & 0.74 & 0.67 & 0.81 & 0.58 & 0.70 & 0.65 & 0.60 & - \end{array}$

**Table 3:** A priori probabilities and probabilities of message reception for actors with evidence $v_{2,11}$ and $v_{2,20}$.

$\begin{array}{cccccccccccc} & v_{1,1} & v_{1,2} & v_{1,3} & v_{1,4} & v_{1,5} & v_{1,6} & v_{1,7} & v_{1,8} & v_{1,9} & v_{1,10} & \ldots \\ A\;\mathrm{priori} & 0.68 & 0.52 & 0.59 & 0.60 & 0.87 & 0.76 & 0.76 & 0.87 & 0.91 & 0.66 & \ldots \\ \mathrm{Ev}.v_{2,11} & 0.88 & 0.77 & 0.74 & 0.79 & 0.89 & 0.89 & 0.90 & 0.94 & 0.90 & 0.84 & \ldots \\ \mathrm{Ev}.v_{2,20} & 0.81 & 0.68 & 0.69 & 0.73 & 0.88 & 0.84 & 0.85 & 0.91 & 0.90 & 0.78 & \ldots \\ & v_{1,11} & v_{1,12} & v_{1,13} & v_{1,14} & v_{1,15} & v_{1,16} & v_{1,17} & v_{1,18} & v_{1,19} & v_{1,20}\\ A\;\mathrm{priori} & 0.39 & 0.91 & 0.73 & 0.66 & 0.81 & 0.57 & 0.70 & 0.65 & 0.59 & 0.92\\ \mathrm{Ev}.v_{2,11} & 0.71 & 0.96 & 0.89 & 0.94 & 0.92 & 0.81 & 0.86 & 0.84 & 0.78 & 0.94\\ \mathrm{Ev}.v_{2,20} & 0.54 & 0.93 & 0.84 & 0.79 & 0.87 & 0.79 & 0.80 & 0.85 & 0.73 & 0.92 \end{array}$

## Appendix A

Table A1 contains all conditionals from directors to institutions. Those from institutions to directors are not shown, but can be deduced from the affiliation matrix in Table A1, in conjunction with the fact outlined earlier that institutions will send messages to directors, always with a transfer probability 0.9.

**Table A1:** Conditionals of the Directors network from actors to institutions.

V252=1∣V112=1[0.76]V215=1∣V112=1[0.76]V218=1∣V112=1[0.76]V221=1∣V112=1[0.76]V222=1∣V112=1[0.76]V252=1∣V122=1[0.91]V292=1∣V122=1[0.91]V210=1∣V122=1[0.91]V217=1∣V122=1[0.91]V222=1∣V122=1[0.91]V212=1∣V132=1[0.90]V212=1∣V132=1[0.90]V212=1∣V142=1[0.87]V212=1∣V142=1[0.87]V221=1∣V142=1[0.87]V292=1∣V152=1[0.58]V213=1∣V152=1[0.58]V232=1∣V162=1[0.70]V292=1∣V162=1[0.70]V221=1∣V162=1[0.70]V222=1∣V172=1[0.70]V232=1∣V172=1[0.70]V262=1∣V172=1[0.70]V292=1∣V172=1[0.70]V221=1∣V172=1[0.70]V222=1∣V172=1[0.70]V252=1∣V182=1[0.61]V272=1∣V182=1[0.61]V210=1∣V182=1[0.61]V217=1∣V182=1[0.61]V218=1∣V182=1[0.61]V221=1∣V182=1[0.61]V223=1∣V182=1[0.61]V211=1∣V192=1[0.54]V215=1∣V192=1[0.54]V221=1∣V192=1[0.54]V222=1∣V192=1[0.54]V222=1∣V110=1[0.76]V262=1∣V110=1[0.76]V292=1∣V110=1[0.76]V221=1∣V110=1[0.76]V222=1∣V110=1[0.76]V223=1∣V110=1[0.76]V272=1∣V111=1[0.99]V211=1∣V111=1[0.99]V215=1∣V111=1[0.99]V217=1∣V111=1[0.99]V219=1∣V111=1[0.99]V222=1∣V111=1[0.99]V252=1∣V112=1[0.57]V272=1∣V112=1[0.57]V211=1∣V112=1[0.57]V215=1∣V112=1[0.57]V219=1∣V112=1[0.57]V221=1∣V112=1[0.57]V222=1∣V112=1[0.57]V212=1∣V113=1[0.70]V292=1∣V113=1[0.70]V210=1∣V113=1[0.70]V213=1∣V113=1[0.70]V214=1∣V113=1[0.70]V216=1∣V113=1[0.70]V219=1∣V113=1[0.70]V221=1∣V113=1[0.70]V222=1∣V113=1[0.70]V292=1∣V114=1[0.77]V211=1∣V114=1[0.77]V215=1∣V114=1[0.77]V216=1∣V114=1[0.77]V221=1∣V114=1[0.77]V222=1∣V114=1[0.77]V292=1∣V115=1[0.65]V215=1∣V115=1[0.65]V222=1∣V115=1[0.65]V242=1∣V116=1[0.85]V282=1∣V116=1[0.85]V292=1∣V116=1[0.85]V217=1∣V116=1[0.85]V218=1∣V116=1[0.85]V220=1∣V116=1[0.85]V221=1∣V116=1[0.85]V222=1∣V116=1[0.85]V224=1∣V116=1[0.85]V212=1∣V117=1[0.77]V292=1∣V117=1[0.77]V212=1∣V117=1[0.77]V242=1∣V118=1[0.81]V282=1∣V118=1[0.81]V219=1∣V118=1[0.81]V220=1∣V118=1[0.81]V224=1∣V118=1[0.81]V242=1∣V119=1[0.87]V292=1∣V119=1[0.87]V214=1∣V119=1[0.87]V252=1∣V120=1[0.56]V211=1∣V120=1[0.56]V219=1∣V120=1[0.56]V221=1∣V120=1[0.56]V222=1∣V120=1[0.56]
